# An Overview of Molecular Mechanism, Clinicopathological Factors, and Treatment in NUT Carcinoma

**DOI:** 10.1155/2019/1018439

**Published:** 2019-11-11

**Authors:** Qian W. Huang, Li J. He, Shuang Zheng, Tao Liu, Bei N. Peng

**Affiliations:** ^1^Department of Medical Oncology, People's Hospital of Boluo County, Huizhou 516000, China; ^2^Department of Medical Oncology, People's Hospital of Liaoning Province, Shenyang 110000, China; ^3^Department of Orthopaedics, People's Hospital of Gaotang County, Liaocheng 252000, China; ^4^Dalian Medical University, Dalian 116000, China

## Abstract

NUT carcinoma (NC) is a rare and poorly differentiated tumor, with highly aggressive and fatal neoplasm. NC is characterized by chromosomal rearrangement involving NUTM1 gene, but lack of specific clinical and histomorphological features. It is more common in midline anatomic sites, such as head and neck, mediastinum, and other midline organs. NC may occur at any age, but mainly in children and young adults. In addition, male and female are equally affected. Most clinicians lack a clear understanding of the disease, and NC diagnostic reagents are still not widely used; therefore, misdiagnosis often occurs in clinic. Due to the highly aggressive nature of the disease and the insensitivity to nonspecific chemotherapy or radiotherapy, many patients have died before the confirmation of NC. In fact, the true incidence of NC is much higher than the current statistics. In recent years, targeted therapy for NC has also made some progress. This article aims to summarize the molecular mechanisms, clinicopathological characteristics, and treatment of NC.

## 1. Progress of NUT Carcinoma

NUTM1 (NUT midline carcinoma family member 1, aka NUT) gene, on chromosome 15, is normally expressed only in mature spermatogonia and has no known function [1]. NUT carcinoma (NC), a rare and poorly differentiated tumor, is characterized by chromosomal rearrangement involving NUT gene, without any clinical or histomorphological features to distinguish it in clinical diagnosis [2]. In 1991, NC was first described in two cases, characterized by t(15; 19) translocation [3, 4]. In 2003, scholars found that the occurrence of t(15; 19)(q13; p13.1) translocation caused the formation of a BRD4-NUT fusion oncogene [5]. In most of the previous cases, NC arose from midline anatomic structures, such as the head, neck, and mediastinum [6, 7]. In 2004, NC was defined as midline carcinoma with NUT rearrangement, also called NUT midline carcinoma, which was caused by NUT gene on chromosome 15 fused to BRD4 gene on chromosome 19 or other fusion partner genes, leading to the formation of BRD4-NUT fusion oncogene or NUT-variant fusion oncogene [8, 9]. However, more and more studies have found that NC arose not only in midline structures but also in the lung [10], pancreas [11], kidney [12], bladder [8], endometrium [8], salivary gland [13], bone [14], ovarian [15], and other organs or soft tissues.

Therefore, the WHO classification of tumors removed the word “midline” from the name of this type of tumors and redefined it as NUT carcinoma in 2015 [15].

## 2. Genetic Abnormality of NUT Carcinoma

Somatic cytogenetic abnormality is the basis of NC. Cytogenetic analysis shows that the oncogene of NC includes the rearrangement of the NUTM1 gene with a set of partner genes, mainly fused to the paralogous genes encoding bromodomain and extraterminal domain proteins (BET proteins), including BRD2, BRD3, BDR4, and BRDT [16–18]. In two-thirds of the cases, NUT gene is fused to BRD4 resulting in BRD4-NUT fusion gene [19]. BRD3 [20] and NSD3 [21] are also relatively common fusion partners with NUT. Recently, accumulating studies have identified novel fusion partners, including ZNF532 [22], ZNF592 [23], MXD4 [24], BCORL1 [25], MXD1 [15, 25], CIC [26], MGA [27], and other unknown genes.

## 3. Pathogenic Mechanism

NC is a highly invasive tumor driven by NUT fusion oncoprotein. The normal single molecule of NUT, the family of nuclear protein in testis, has two acidic domains (AD), and one of which binds to histone acetyltransferase (HAT) p300, resulting in histone acetylation [28]. The most common NUT fusion partners are the members of BET family, which is a special protein family of transcription/chromosome regulators, including BRD2, BRD3, BDR4, and BRDT, and the single protein molecule of all members contains two bromodomains and an extraterminal (ET) domain [29]. BRD2, BRD3, and BRD4 are widely expressed in organs, while BRDT is limited to the testis [30]. As a key member of the BET family, BRD4 plays an important role in regulating transcription, cell growth, cell cycle, and chromatin structure and its dysregulation is associated with many tumors [31–36]. The BRD4 bromodomains can specifically recognize and bind acetylated lysine residues of histone and other proteins, and the ET domain can bind to a series of chromatin-modifying proteins as the protein-protein interaction module [17]. The BRD4-NUT fusion oncoprotein retains the bromodomains and ET domain of BRD4 and nearly complete the coding sequence of NUT. In vitro cell studies showed that knockdown of the BRD3/4-NUT gene by siRNA in NC cell lines induced rapid squamous differentiation and arrested growth, which suggested that the BRD3/4-NUT fusion protein blocked differentiation and promoted proliferation of carcinoma cells [20]. Therefore, the mechanism of BRD-NUT oncoprotein is to restrict cell differentiation and promote uncontrolled cell growth.

The interaction of acetylated lysine residues with bromodomains is pivotal for the carcinogenic function of BRD4-NUT fusion protein [37]. BRD4-NUT protein is contained in huge nuclear foci produced by combining BRD4-NUT with acetylated chromatin through acetylated lysine residues on histone [28]. Some scholars analyzed the nuclear foci of BRD4-NUT in NC cell lines, and the results showed that BRD4-NUT was highly enriched in adjacent regions of acetylated chromatin. In NC cell lines, BRD4 bromodomains can combine with histone acetylated lysine residues which promote the binding of BRD4-NUT to chromatin and produce foci of BRD4-NUT and acetylated chromatin. The NUT component of BRD4-NUT complexes can recruit p300, leading to the high level of local histone acetylation, further producing BRD4-NUT complexes in a feed-forward mechanism. Finally, it causes the formation of the huge regions containing acetylated chromatin, BRD4-NUT, and EP300, and the huge regions are termed “megadomains” as the whole topologically associating domains (TADs) can be filled with acetylated chromatin and BRD4-NUT oncoprotein [23, 38]. The resultant megadomains cover the regulatory regions of MYC and p63, both of which have been proved to be necessary for the growth of NC cell lines. After the knockdown of the MYC or p63 in NC cell lines, cell growth stopped, especially in case of MYC knockdown, which also led to cell differentiation [39]. This indicated that MYC and p63 are the key target genes of BRD4-NUT. Thus, BRD4-NUT might directly misregulate these two key genes, driving the occurrence of NC. The pathogenic mechanism of NSD3-NUT [21] and ZNF532-NUT [22] fusion proteins is similar to that of BRD4-NUT.

In addition, a recent study showed BRD4 was hyperphosphorylated in NC, and CDK9 was the potential kinase mediating BRD4 hyperphosphorylation. Blocking BRD4 hyperphosphorylation with chemical and molecular inhibitors, the expression of BRD4 downstream oncogenes was inhibited and cell transformation was abrogated [38]. It suggested that BRD4 hyperphosphorylation was associated with its function to drive the expression of downstream oncogenes and cellular transformation in NC.

## 4. Clinicopathological Features and Diagnosis of NUT Carcinoma

At present, the cellular origin of NC is still unclear. According to previous reports, NC might be derived from the malignant epithelial tumor, while there were rare reports, suggesting it might be originated from mesenchymal cells [27, 40]. Nothing is certainly known about the etiology of NC, which was found to be not associated with Epstein–Barr virus (EBV) and human papillomavirus (HPV) infection [41], and also different from some squamous cell carcinomas closely related to environmental factors. Although the diagnosed cases of NC have been increasing in recent years, its actual incidence remains unknown.

NC lacks specific clinical manifestations and histomorphological features. It is usually found in the midline anatomic sites, such as the head, neck, or thorax, and also it is easy to report NC as diagnosed in other tissues or organs. It can occur at any age, ranging from newborn to 78 years, but mainly in children and young adults. In addition, male and female are equally affected [42, 43]. NC is a fatal disease with extremely poor prognosis, and most patients died within a year after diagnosis. In 2012, a retrospective study [18] of 63 NC patients revealed that the median age of the patients was 16 years (range 0–78 years). About 56% of all patients had the tumor occurred in the thorax and 21% in the head and neck. The median overall survival (OS) of patients with NC was 6.7 months, and the one-year OS was 30%. A recent large cohort study (*n* = 119) [44] reported that the median age of NC patients was 23 years (range 0–68 years). The majority of tumors arose in the lung (35.3%), head and neck (35%), and mediastinum (26%). The median OS was only 5 months, and the one-year OS was 24.99%. Both of the two studies revealed equal incidence in males and females. Although an earlier study showed the average survival time of NC patients with NUT-variant was increased almost fourfold compared to that of the patients with BRD4- NUT [8], it was also reported that the patients with NUT-variant had no recurrence within about 34 months after surgery [45]. However, in the two large cohort studies mentioned above, both of them failed to show significant difference in OS among translocation types (BRD4-NUT, BRD3-NUT, and NUT-variant) [18, 44]. Therefore, further cohort studies are required to investigate whether the prognosis of NUT-variant NC patients is better than that of BRD3/4-NUT patients, which may contribute to identify molecular subtypes with unique prognostic features.

The histopathology of NC is not usable for diagnosis due to lack of typical morphological characteristics. The more common description is a poorly differentiated or undifferentiated carcinoma with abrupt and focal squamous differentiation, containing medium-sized round or oval cells with high nuclear-to-cytoplasm ratio, with variably prominent nucleoli and pale cytoplasm. Thin rim and foci of abrupt keratinization are often present [46, 47]. NC have also been reported to display different appearances, including high-grade spindle cell neoplasms [48], small round blue cell sarcoma [24], and high-grade neuroepithelial neoplasm with PNET [27]. Because the histopathological features of NC overlap with other poorly differentiated/undifferentiation tumors or appear similar to several commonly seen pathologies, it often leads to misdiagnosis. It is significant to remind the clinicians to reconsider some cases of diagnosed squamous cell carcinoma, germ cell tumors, neuroendocrine carcinoma, and small round blue cell sarcoma for NC differential diagnosis [46, 49].

NC was initially diagnosed by the use of fluorescence in situ hybridization (FISH) and reverse-transcriptase polymerase chain reaction (RT-PCR), which directly detects the NUT gene rearrangement. In 2009, a specific monoclonal antibody against NUT (C52B1) was developed for the diagnosis of NC and had the specificity of 100% and sensitivity of 87% [50]. If immunohistochemical (IHC) nuclear staining was observed as more than 50%, it could confirm the diagnosis of NC [51]. Although the C52B1 can be directly used for diagnosis of NC, the mutation subtypes such as BRD4-NUT, BRD3-NUT, or NUT-variant cannot be identified. Thus, FISH, RT-PCR, and next-generation sequencing (NGS) are still necessary to determine the gene fused to NUT. With the development of targeted therapy, it is important to identify the specific genetic subtype of NC.

## 5. Therapy Strategies

There is no constantly effective treatment strategy for NC to date. It was reported that radiotherapy and surgical resection could prolong progression-free survival (PFS) and OS for NC patients, but chemotherapy had nothing to do with improved outcome [18]. Another study showed that chemotherapy and radiotherapy were associated with the higher survival rate, but not applicable to surgical resection [44]. A 10-year-old boy [14] with NC involving the iliac bone was initially diagnosed as Ewing sarcoma and received the SSG-IX protocol and local radiotherapy. This patient has remained in complete remission for 13 years. Similar therapy regimens have been used in NC patients before, but the outcome was not satisfactory. Therefore, the effect of surgery, chemotherapy, or radiotherapy on the prognosis of NC patients is still not clear because relevant data are obviously lacking. Although some NC patients have showed response to chemotherapy or radiotherapy, in most cases, the time of remission was short, and then the patients relapsed and died soon.

Targeted therapy has become focus on the clinical research of NC therapy. Histone deacetylase inhibitors (HDACi) and BET inhibitors (BETi) are target drugs against NC which were firstly found. HDACi was proved to significantly inhibit tumor cell growth and to induce differentiation in NC cell lines and murine xenograft models of NC [52]. Based on research findings, a 10-year-old boy [45] with NC was treated with a single agent of HDACi vorinostat and showed significant response after five weeks of therapy. Due to severe (grade 3) nausea and emesis, this patient stopped to receive the treatment of vorinostat, and then the tumor grew rapidly. He died with an OS of 11 months. BETi is an acetyl-histone mimetic compound, which can bind to bromodomains and competitively inhibit the tether of BRD3/4 to acetylated chromatin, and directly target BRD3/4-NUT fusion protein. In 2010, a study [53] found that BETi JQ1 could induce differentiation and inhibit growth of NC cells in vivo and in vitro. Because of significant preclinical response of BETi, phase I/II clinical trials for the safety and efficacy of different BETis in NC patients are currently under way. The clinical efficacy of BETi in 4 patients with NC has been reported in 2016 [54]. Two of them showed a rapid response with tumor regression, and one maintained disease stabilization. The OS of 4 cases was 19, 18, 7, and 5 months, respectively. Compared with previously described NC patients with 6.7 or 5 months of median OS, it proved that HDACi and BETi could significantly prolong the survival of NC patients. Interestingly, Stirnweiss and his colleagues [55] found that the BETi was more sensitive in BRD4-NUT (ex11:ex2) variant NC cell lines than in BRD4-NUT (ex15:ex2) variant or non-NC cell lines. The BETi was also effective in the BRD3-NUT fusion cell line. The result suggested that different breakpoints or fusion subtypes in NC tumors might have different responses to BETi. This indicated that BETi had the possibility of ineffective treatment and reminded the researchers of the necessity to identify fusion gene for the decision of specific NC therapy with maximized effectiveness. However, the efficacy of HDACi and BETi is limited by drug toxicity such as the unwanted effect on normal cells, and BETi is also limited by the acquisition of resistance [56, 57]. Therefore, to a large extent, the effective and precise treatment of NC has become more difficult.

Recently, scholars revealed that a novel dual HDAC/PI3K inhibitor (CUDC-907) showed the strongest outcomes on NC cells in vitro compared to HDACi or BETi [58, 59]. The mechanism of CUDC-907 is to downregulate MYC expression and inhibit the growth of MYC-driven malignant cells by targeting the upstream regulators of MYC, such as BRD4-NUT and phosphoinositide 3-kinases (PI3K) [60]. Thus, CUDC-907 might be a promising target drug for NC therapy. In addition to HDACi, BETi, and HDAC/PI3K inhibitors, CDK9 inhibitors [61] and mTOR inhibitors [62] in drug screening were proved to be sensitive drugs against NC in vitro. Both of them also showed remarkable efficacies to inhibit the proliferation of NC cells.

## 6. Conclusion

NC is a rare and highly lethal carcinoma, which lacks special clinicopathological features. While IHC, FISH, RT-PCR, and NGS are still not widely used for the diagnosis of NC and clinicians lack understanding about the disease, NC can be easily misdiagnosed. Early recognition of NC is crucial to select and establish the optimal treatment regimens. Great progress has also been made in the development of NC therapy, especially targeted therapies, which shows a promising tendency. Today, it is clear that NC is no longer confined to the midline structure, and it can occur in any tissue or organ, at any age. What we are facing now is not only to help clinicians to raise their awareness of NC but also to clarify the criteria for when to consider NC. The diagnosis of a poorly or undifferentiated carcinoma should prompt clinicians to consider the possibility of NC, and small round cell sarcoma, neuroendocrine carcinoma, germ cell tumors, and Ewing sarcoma/PNET are also taken into account to initiate NC differential diagnosis. The previously hidden and currently increasing occurrence of NC make the clinicians and patients strive for early detection of NC and timely symptomatic treatment, as well as more advanced target anti-NC therapy.
